# Hospital Progress and Finance

**Published:** 1923-10

**Authors:** 


### HOSPITAL PROGRESS AND FINANCE.

### THE SWINDON CRISIS: AN EXPERT VIEW.

MR. RODEN ORDE, who, it will be remembered, left the Royal Victoria Infirmary, Newcastle-on-Tyne, to work for the British Red Cross Society, where he assisted Sir Napier Burnett in the latter's investigation of the financial condition of the Royal Sussex County Hospital, Brighton, has now himself issued a special report on the hospital needs of Swindon. The Great Western Railway Medical Benevolent Fund Society has long desired another hospital on a new site, and Mr. Orde has been investigating the matter. We recently discussed the serious situation, in the hospital sense, at Swindon, and Mr. Orde's first admission is one of its gravity. He suggests a conference of all the interests involved, leading to the appointment of a joint committee on whose report any action should be based, though he hopes it might recommend a joint hospital and joint action. The railwaymen need, and insist upon, hospital treatment for their sick, and if a general agreement were come to, then he thinks the directors of the G.W.R. might be asked to advance the capital for building the hospital on the Park site. Of the 90,000 inhabitants 40,000 are railwaymen. Mr. Orde points out that 150 beds, in place of the present 44, are needed. The Victoria Hospital extension, now in progress, is mainly administrative and will add only six beds at once, but its site of 1¼ acres is too small to be conveniently that of a large hospital. The railwaymen's hospital, close to the works, has twelve beds only. Mr. Orde's final conclusion is that Swindon needs one hospital for its whole population, and his considered opinion is based on so wide an experience that it should be regarded with the utmost respect.

### A STEP FORWARD AT KING'S.

LORD HAMBLEDON'S announcement that another (the fourth) of the wards now closed at King's College Hospital for want of money, is being reopened for paying patients at a fee of four and a half guineas a week is significant. While these wards cannot be maintained, at present cost, by the hospital's own revenue, it is well that they should be used for the middle-class patient who is no less in need of aid than his poorer sick neighbour. Criticism could be made of the plan only if there were enough money to keep the wards open for the free treatment and maintenance of the destitute. Indeed the plan, according to American experience, may even bring the time nearer when these wards can revert to their original purpose, for the middle-class paying patient often becomes a zealous supporter who remembers his hospital either by an annual subscription after leaving or in his will, or both. If and when this experience is repeated here, the time will have come for a regular and additional block devoted to the paying patient permanently. Moreover, many such patients are being maintained, we are told, by congregations and other bodies interested in their welfare, and this collective support, being disinterested in motive, often itself endures after its occasion has passed. Whatever sum we may contribute to a hospital, while an in-patient, we are all recipients of charity, for charity begot our hospitals and still maintains them. The new plan widens the field from which this generosity springs, and is therefore congenial to the voluntary spirit. It is a step toward that grading of wards and patients, from the necessitous to the wealthy, which a completed voluntary system would secure.

### THE PAYING "PAUPER."

ONCE again the anomalous legal position of the paying patient who accepts treatment at a Poor Law Infirmary is exposed. A member of the Kingston Board of Guardians has thereby become technically "a pauper," at considerable expense, and has been legally deprived of his vote upon the board. Last time a poor fellow whose relations had paid for his treatment was deprived of his pension: now a well-to-do ratepayer is stripped of his office and his vote. The law, in this matter, deals out injustice with calm impartiality, and at least gives the latter no advantage. Incidentally it places the Ministry of Health in the ridiculous position of urging, on the one hand, paying patients to make use of the infirmaries for their own sake and to relieve the pressure on voluntary hospital beds, whilst on the other it gives legal denial to the Ministry's assertion that the pauper taint has been removed. No wonder Parliamentary government falls into contempt so long as it allows such anomalies to continue unreformed.

### THE FIRST REPORT FROM SHEFFIELD.

THE remarkable first annual report of the Sheffield Joint Hospitals' Council is that which every informed person expected it to be—a record, in the main, of a great success, not yet in full accomplished. Having regularly recorded the steps by which the Council launched the penny-in-the-pound scheme, and the activities of Mr. Lamb, its ardent advocate and organising secretary, we must content ourselves with a grateful glance at the results that these united efforts have attained. Because they justify themselves they are their best praise and comment. Though the Council's work and the penny-in-the-pound scheme overlap to some extent, justice can be done to both only by regard to their respective in- or, rather, interdependence. For instance, the Council has endeavoured, in the face of intelligible difficulties, to co-ordinate the hospitals of the district with Sheffield; and the penny scheme has found equally natural obstacles in the way of its advance toward the rural areas. Upon local and central financial schemes it is wiser not to adjudicate in general, but to seek fairplay to both in each case where they meet or cause fears of overlapping. Suffice it, summarily, to say that the association of contributors already numbers 153,000 members; that the Council tries to omit no agency, from the old Hospital Sunday movement to the new Cinema Day organisation, from its co-operative endeavours; and that aftercare, motor ambulances and convalescent treatment engage its thoughts. The Local Committee, suggested in each area by the Voluntary Hospitals Commission, is also dovetailed into the work; health lectures, as we hoped when they began, have become a recurring series; and the Council has recommended that "letters" or recommendations should be waived on behalf of the unemployed in search of hospital treatment. The total collected has been over £62,000, and seems to be growing in the present year; £52,000 odd has been distributed to hospitals; and expenses have been £10,000, or one-sixth of the receipts. The report has the pretension of a blue-book, but is illustrated, better printed and more interesting than most to read. As to the future, a new general hospital is contemplated when a site shall have been found. More beds and more money are Sheffield's two great needs, and the growth of income may be able to supply them.

### FOILING SECRESY.

AFTER the Lewisham Board of Guardians had decided not to publish the report of the late public inquiry, the Ratepayers' Association of the borough communicated with the Ministry of Health, who returned a comprehensive summary of the conclusions from the report of Mr. J S. Oxley, General Inspector, issued after the official inquiry that he had held. Since the Guardians have acted on the recommendations that the inspector's conclusions involved—dismissed the hospital steward, and reinstated the assistant steward, for example—it suffices to record that in the considered opinion of the Ministry, the conclusions, at least, of a Report resulting from a public inquiry are public property, and that the ratepayers have a recognised means of ensuring this, should an attempt be made to withhold them. The mere suggestion that a report of a not dissimilar kind should not be published brought a large South Coast voluntary hospital temporarily into bad repute the other day. The old principle, therefore, is re-established that both voluntary and municipal institutions should be conducted and, if necessary, investigated in the open. Any breach of this principle recoils at once upon the management concerned.

### BRADFORD'S BIG SCHEME.

BEFORE the war a site for a new infirmary at Bradford was purchased at Daisy Hill, and plans were prepared. The cost was to be found partly by private subscriptions and partly by the sale for £100,000 of the present infirmary and site. This last was the offer made by the Corporation in 1914. If this offer still holds good—and this will not be known definitely till after the REVIEW appears—the board of the present infirmary, the advisory council and the new infirmary committee have unanimously decided to proceed with the new infirmary scheme. It has also been decided to start a fund for erecting the proposed new building. Should the offer be renewed and sufficient funds be available, an ambitious and important scheme will have been launched which even before the war was considered necessary.

### A REAL LEAD FROM LEEDS.

OF 4,133 firms in the City of Leeds, grouped in twenty blocks of buildings, 566 only subscribed last year to the four voluntary hospitals there. To include all the firms, as is gradually being accomplished at Sheffield, Mr. T. F. Braime has started a scheme. The well-wishers in each block are asked to place and maintain the hospital's needs before the other occupiers in the hope that they will become annual subscribers. The General Infirmary has an overdraft of over £54,000; the other hospitals are proportionately in debt; and all four have united to form a Leeds Voluntary Hospitals Fund, for which regular annual support is being invited. Mr. Braime's latest scheme contemplates organising the professions and distributive or retail trades as the workshops and factories and the employers have already been organised. The Workpeople's Fund collects some £30,000 a year; the Employers' Contribution Fund raises at present some £7,000 a year from 700 regular members. The new fund would grade subscriptions somewhat as follows—from big banks and companies, say, fifty guineas; from professional men, ten to twenty; from shops in side streets, one guinea; from the lowest-paid staffs, not already contributors to the Workpeople's Fund, twopence a week. One hundred firms are stated to have blessed this scheme before it was made public, and their promises amount to £1,665 under the scheme. Only half as much again was subscribed last year from the entire city! If this section can be organised successfully, the organisation of Sectional Subscriptions, which we have discussed and advocated for ten years or so, will be roughly accomplished.

### THE RE-NAMING OF WARDS.

MR. FRANK GREEN raises a timely point in the September number of St. Bartholomew's Hospital *Journal*. Apparently "John" Ward has been re-christened, or rather re-named, "Smithfield," because of a generous gift from the members of the market. Mr. Smith inquires if the secularisation of ward names by substituting those of present benefactors for those of patron saints is altogether happy. He asks further where it may lead? Finally he suggests that it tends towards nationalisation, because the Church, which is the guardian of the Christian tradition of private charity and, in consequence of private property, is thus set aside as the inspiration of its voluntary system. We have our own views upon this question, but for the moment we stand aside to invite our readers opinions.

### PATIENTS' FRIENDS.

A HOSPITAL secretary has lately described in the *Daily News* the flood of visitors that called upon a patient upon the first visiting day, the falling off in numbers that ensue dafterwards, and the patient's final departure with no one but a nurse to see her off. This admission is of more than passing interest, since the visiting of patients raises many problems for hospital managers, and yet the one who recounts the experience did so only to complain that there had not been more visitors. Would the same motive have been found in a State-hospital official?

